# Development and web deployment of prediction model for pulmonary arterial pressure in chronic thromboembolic pulmonary hypertension using machine learning

**DOI:** 10.1371/journal.pone.0300716

**Published:** 2024-04-05

**Authors:** Takaaki Matsunaga, Atsushi Kono, Mizuho Nishio, Takahiro Yoshii, Hidetoshi Matsuo, Mai Takahashi, Takuya Takahashi, Yu Taniguchi, Hidekazu Tanaka, Kenichi Hirata, Takamichi Murakami

**Affiliations:** 1 Department of Radiology, Kobe University Graduate School of Medicine, Kobe, Japan; 2 Kobe University School of Medicine, Kobe, Japan; 3 Division of Cardiovascular Medicine, Department of Internal Medicine, Kobe University Graduate School of Medicine, Kobe, Japan; University of Alabama at Birmingham, UNITED STATES

## Abstract

**Background and purpose:**

Mean pulmonary artery pressure (mPAP) is a key index for chronic thromboembolic pulmonary hypertension (CTEPH). Using machine learning, we attempted to construct an accurate prediction model for mPAP in patients with CTEPH.

**Methods:**

A total of 136 patients diagnosed with CTEPH were included, for whom mPAP was measured. The following patient data were used as explanatory variables in the model: basic patient information (age and sex), blood tests (brain natriuretic peptide (BNP)), echocardiography (tricuspid valve pressure gradient (TRPG)), and chest radiography (cardiothoracic ratio (CTR), right second arc ratio, and presence of avascular area). Seven machine learning methods including linear regression were used for the multivariable prediction models. Additionally, prediction models were constructed using the AutoML software. Among the 136 patients, 2/3 and 1/3 were used as training and validation sets, respectively. The average of R squared was obtained from 10 different data splittings of the training and validation sets.

**Results:**

The optimal machine learning model was linear regression (averaged R squared, 0.360). The optimal combination of explanatory variables with linear regression was age, BNP level, TRPG level, and CTR (averaged R squared, 0.388). The R squared of the optimal multivariable linear regression model was higher than that of the univariable linear regression model with only TRPG.

**Conclusion:**

We constructed a more accurate prediction model for mPAP in patients with CTEPH than a model of TRPG only. The prediction performance of our model was improved by selecting the optimal machine learning method and combination of explanatory variables.

## Introduction

Chronic thromboembolic pulmonary hypertension (CTEPH) is a disease with a poor prognosis caused by stenosis and occlusion of the pulmonary artery due to an organizing thrombus in the pulmonary artery that ultimately leads to pulmonary hypertension (PH) [1, 2]. PH is defined as a mean pulmonary artery pressure (mPAP) of > 20 mmHg on right heart catheterization (RHC).

The mPAP is one of the key indices used for the diagnosis, severity classification, prognosis prediction, and indications for surgery in CTEPH [3, 4]. It is measured using RHC, which is expensive, invasive, and can cause adverse events, including infection, bleeding from local puncture sites, and other morbidities [5]. Echocardiography, computed tomography, and magnetic resonance imaging have been used to estimate mPAP [6]. Tricuspid valve pressure gradient (TRPG) measured using the Doppler method with echocardiography is a well-known method for estimating mPAP [7].

Echocardiography is frequently used in clinical practice because of its simplicity and low cost compared with computed tomography, magnetic resonance imaging, and RHC. However, echocardiography alone is not accurate in predicting mPAP [1, 8, 9]. An accurate, minimally invasive, simple, and inexpensive method to predict mPAP is therefore needed.

In recent years, artificial intelligence such as machine learning and deep learning has been used in various areas of clinical medicine [10, 11]. By using machine learning and deep learning, it is possible to build classification and prediction models from multiple clinical data. To the best of our knowledge, few studies have used machine learning to build mPAP prediction models.

This study compares a conventional method using TRPG with machine learning models to predict mPAP of CTEPH. The hypothesis of this study is whether a low-invasive, high-accuracy assessment and prediction method can be developed using machine learning. For this, we used echocardiography, as well as basic information such as patient age and sex, chest radiography, and blood tests in the model construction. The contributions of this study are as follows: (i) A multivariable prediction model of mPAP with higher accuracy than that of echocardiography alone was constructed by combining multiple minimally invasive tests. (ii) Various machine learning methods were compared to construct the prediction models, and the most accurate method was selected. (iii) The prediction model for the current study can be easily used on our website.

## Material and methods

This retrospective study was approved by the institutional review board (IRB) of Kobe University Hospital (number: B210112). The requirement for informed consent was waived by IRB of Kobe University Hospital. This study conformed to the Declaration of Helsinki and the Ethical Guidelines for Medical and Health Research Involving Human Subjects in Japan (https://www.mhlw.go.jp/file/06-Seisakujouhou-10600000-Daijinkanboukouseikagakuka/0000080278.pdf).

### Subjects

Between April 28, 2005, and January 27, 2020, 167 patients diagnosed with CTEPH at Kobe University Hospital were included in the current study, for whom mPAP was measured by RHC. Of the 167 patients, those with missing values were excluded, and data from 136 patients were used to construct the prediction model of mPAP.

### Data

The following patient data were used as explanatory variables of the prediction model: basic patient information (age and sex), blood tests (brain natriuretic peptide (BNP)), echocardiography (TRPG), and chest radiography (cardiothoracic ratio (CTR) = length of heart/that of thorax, right second arc ratio (RSR) = length of right second arc/that of thorax, and presence of avascular area). The patient data were collected near the date of RHC. The average interval between RHC and TRPG and that between RHC and BNP were 5.8 and 5.2 days, respectively. CTR and RSR, correlated with pulmonary hypertension [12, 13], were calculated as the average of the measurements of the two radiologists (3 years of experience), and the presence of an avascular area was determined by their consensus. In cases where the two radiologists could not achieve a consensus, the decision was made by a third radiologist (5 years of experience). The objective variable of the prediction model was the mPAP measured by RHC.

### Data splitting

Among the 136 patients, 2/3 and 1/3 were used as training and validation sets, respectively. Ten different combinations of data splittings for the training and validation sets were obtained by using different random seeds. As shown below, average of model evaluation results obtained from the 10 different data splittings was calculated. These data splittings were performed for selecting the optimal prediction model and the optimal explanatory variables.

### Numerical transformation of explanatory variables

Among the explanatory variables, BNP and CTR were transformed into logarithms due to their non-normality. In addition, after log transformation, data standardization was performed for each explanatory variable using the mean and standard deviation.

### Machine learning method

To build multivariable prediction models, age, sex, BNP, TRPG, CTR, RSR, and the presence of an avascular area were used. Linear regression, k-nearest neighbor (KNN), decision tree, nonlinear support vector regression (SVR), linear SVR, random forest, and eXtreme gradient boosting (XGBoost) [14] were used as machine learning methods for the multivariable prediction models. In addition, prediction models were constructed using automated machine learning (AutoML), a software program that automatically selects optimal machine learning methods. The AutoML software programs used were FLAML [15], TPOT [16], AutoSklearn [17], and AutoKeras [18]. Python (ver. 3.7.6) was used as programming language.

### Selection of explanatory variables

To further improve the prediction accuracy of the models obtained using the optimal machine learning methods, optimal explanatory variables were selected. Optimal explanatory variables were selected from subsets of the seven explanatory variables (age, sex, BNP, TRPG, CTR, RSR, and presence of avascular area). Prediction accuracy was calculated for the models constructed from different combinations of explanatory variables, and the combination of explanatory variables with the highest prediction accuracy was selected.

### Evaluation for selecting optimal model

R squared was used as an indicator of the model prediction accuracy. In addition, root mean squared error (RMSE) and mean absolute error (MAE) were calculated. R squared, RMSE, and MAE were obtained using the following formulas:

$$\text{R}\;\text{squared}=1-\frac{\sum_{\text{i}}{\left({\text{mPAP}}_{\text{m},\text{i}}-{\text{mPAP}}_{\text{p},\text{i}}\right)}^{2}}{\sum_{\text{i}}{\left({\text{mPAP}}_{\text{m}.\text{i}}-\bar{{\text{mPAP}}_{\text{m}}}\right)}^{2}},$$

$$\text{RMSE}=\sqrt{\frac{\sum_{\text{i}}{\left({\text{mPAP}}_{\text{m},\text{i}}-{\text{mPAP}}_{\text{p},\text{i}}\right)}^{2}}{\text{n}}},$$

$$\text{MAE}=\frac{\sum_{\text{i}}\left|{\text{mPAP}}_{\text{m},\text{i}}-{\text{mPAP}}_{\text{p},\text{i}}\right|}{\text{n}},$$

where mPAP_m_ is the measured mPAP, mPAP_p_ is the predicted mPAP, and $\bar{{\text{mPAP}}_{\text{m}}}$ is the mean mPAP_m_. To calculate the indices of prediction accuracy, the model was constructed using the training set, and the indices were calculated from the validation set using the constructed model. The average of the indices obtained from 10 different data splittings was calculated.

This study used R squared, MAE, and RMSE as the evaluation metrics for selecting optimal machine learning models. However, our variable selection was based solely on R squared. Therefore, not only R squared but also MAE and RMSE were used in this study.

### Final model construction

After selecting the machine learning method and combination of explanatory variables, the final prediction model for mPAP was constructed from the data of 136 patients using the best machine learning method and combination of explanatory variables. As the conventional model, a linear regression model with only TRPG was constructed. The optimal final model was then compared with the conventional model (TRPG-only model). To visually compare and evaluate the prediction results, the prediction results of the two prediction models were illustrated with scatter plots and Bland-Altman plots. In addition, Akaike’s information criterion (AIC) and Bayesian information criterion (BIC) were calculated for the two models.

As shown in the Results section, the final prediction model for mPAP was constructed using linear regression. Since the linear regression model was selected and least prone to overfitting, we did not prepare a separate test set.

## Results

Of the 167 patients diagnosed with CTEPH, 31 with missing values were excluded from the current study. Thus, 136 patients diagnosed with CTEPH were included. The median mPAP was 37 mmHg, and the interquartile range (IQR) was 30.8–45.0 mmHg. Seven explanatory variables were used for model construction: age, sex, BNP, TRPG, CTR, RSR, and presence of avascular area. The median age was 69 years (IQR, 62–75 years), the gender distribution was 73.8% male and 27.2% female, and the median TRPG was 57.1 mmHg (IQR, 46.8–71.0 mmHg). Details of the other variables are presented in Table 1.

**Table 1:** Characteristics of patients.

Data		Median or number of patients	(IQR or ratio)
N		136	
age		69	(62–75)
gender			
	male	99	(73.8%)
	female	37	(27.2%)
mPAP (mmHg)		37	(30.8–45.0)
BNP (pg/mL)		85.4	(37.6–299)
TRPG (mmHg)		57.1	(46.8–71.0)
CTR		0.535	(0.495–0.566)
presence of avascular area		14	(10.3%)
RSR		0.170	(0.140–0.193)

Abbreviations: mPAP (mean pulmonary artery pressure), TRPG (tricuspid valve pressure gradient), CTR (cardiothoracic ratio), RSR (right second arc ratio)

To select the optimal machine learning method, linear regression, KNN, decision tree, nonlinear SVR, linear SVR, random forest, and XGBoost were compared. Seven variables were used as explanatory variables to construct the prediction models with these seven machine learning methods. The detailed results of the seven prediction models are listed in Table 2. The best averaged R squared value was 0.360 for the prediction model with linear regression, followed by KNN and linear SVR (R squared, 0.307 and 0.298, respectively).

**Table 2:** Performance evaluation of machine learning method.

Machine learning	R squared	RMSE	MAE
Linear Regression	0.360	0.773	0.634
KNN	0.307	0.811	0.638
Decision Tree	-0.358	1.131	0.866
nonlinear SVR	0.295	0.820	0.632
linear SVR	0.298	0.811	0.629
Random Forest	0.276	0.830	0.636
XGBoost	0.159	0.888	0.682

Abbreviations: SVR (support vector regression), RMSE (root mean square error), MAE (mean absolute error)

Note: Values are obtained by averaging the validation set results over 10 different data splittings.

Additionally, prediction models for mPAP using AutoML were constructed and evaluated (Table 3). The AutoML software programs used were FLAML, TPOT, AutoSklearn, and AutoKeras. Seven explanatory variables were used in AutoML. The averaged R squared for mPAP prediction were as follows: TPOT = 0.348, AutoSklearn = 0.320, AutoKeras = 0.274, and FLAML = 0.178. Thus, among all the prediction models constructed in the current study, linear regression was the most accurate machine learning method.

**Table 3:** Performance evaluation of AutoML.

AutoML	R squared	RMSE	MAE
FLAML	0.178	0.869	0.675
TPOT	0.348	0.782	0.638
AutoSklearn	0.320	0.803	0.633
AutoKeras	0.274	0.823	0.667

Abbreviations: RMSE (root mean square error), MAE (mean absolute error)

Note: Values are obtained by averaging the validation set results over 10 different data splittings.

To select the optimal combination of the explanatory variables, all combinations of the seven variables were compared. Here, linear regression was used as the machine learning method. Table 4 shows the 10 best combinations of explanatory variables and their averaged R squared values. The averaged R squared of the best model was 0.388, and the explanatory variables were age, BNP, TRPG, and CTR. The 2^nd^ and 3^rd^ best R squared values were as follows: the combination of age, BNP, TRPG, CTR, and RSR = 0.384; the combination of age, BNP, and TRPG = 0.377.

**Table 4:** Top-10 optimal linear regression models.

Explanatory variables	R squared
age, BNP, TRPG, CTR	0.388
age, BNP, TRPG, CTR, RSR	0.384
age, BNP, TRPG	0.377
age, BNP, TRPG, CTR, avascular area	0.377
age, BNP, TRPG, CTR, gender	0.375
age, BNP, TRPG, CTR, RSR, gender, avascular area	0.373
age, BNP, TRPG	0.372
age, BNP, TRPG, CTR, RSR, gender	0.372
age, BNP, TRPG, avascular area	0.371
age, BNP, TRPG, gender	0.364

Abbreviations: TRPG (tricuspid valve pressure gradient), CTR (cardiothoracic ratio), RSR (right second arc ratio)

Note: BNP and CTR were transformed into logarithms. Values were obtained by averaging the validation set results over 10 different data splittings.

After the best machine learning method and the best combination of explanatory variables were determined, all data from the 136 patients were used to construct the final optimal model. The conventional linear regression model with only TRPG was compared with the optimal multivariable linear regression model of the current study (the combination of age, BNP, TRPG, and CTR). The R squared of the prediction model was 0.233 for the univariable linear regression model with only TRPG. The R squared of the optimal multivariable linear regression model (0.388) was higher than that of the conventional model. The equation and detailed information of our final model are included in the Supporting Information (Supporting Information, S1 File). The results of the normality or homoscedasticity of the errors in our final model is shown in the Supporting Information (Supporting Information, S1 Fig). S1 Fig shows that there were no significant issues with the normality or homoscedasticity of the errors.

**Fig 1:**
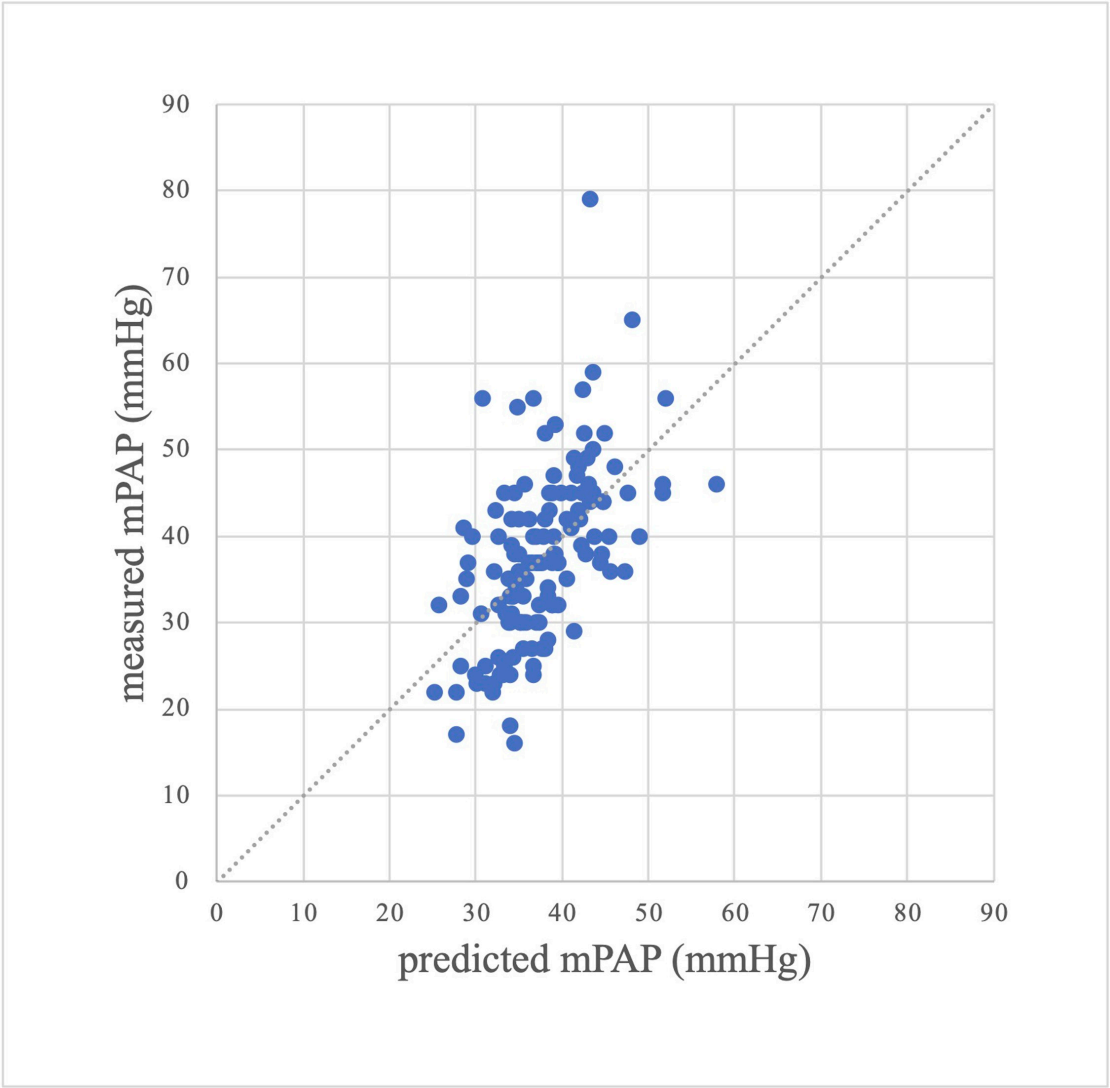
Scatter plot between measured and predicted mPAP (univariable model with only TRPG). Abbreviations: mPAP (mean pulmonary artery pressure), TRPG (tricuspid valve pressure gradient).

**Fig 2:**
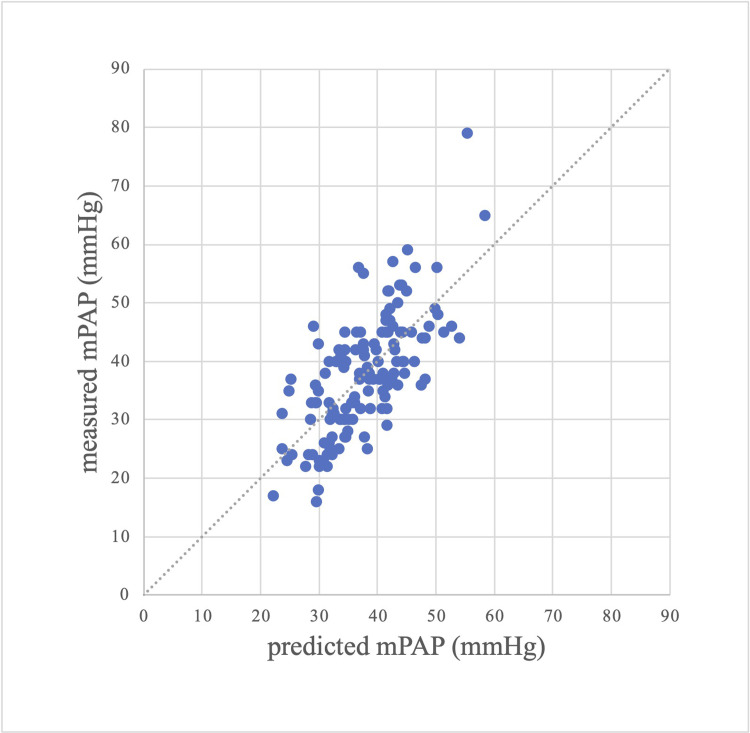
Scatter plot between measured and predicted mPAP (multivariable model with 4 variables). Abbreviation: mPAP (mean pulmonary artery pressure).

**Fig 3:**
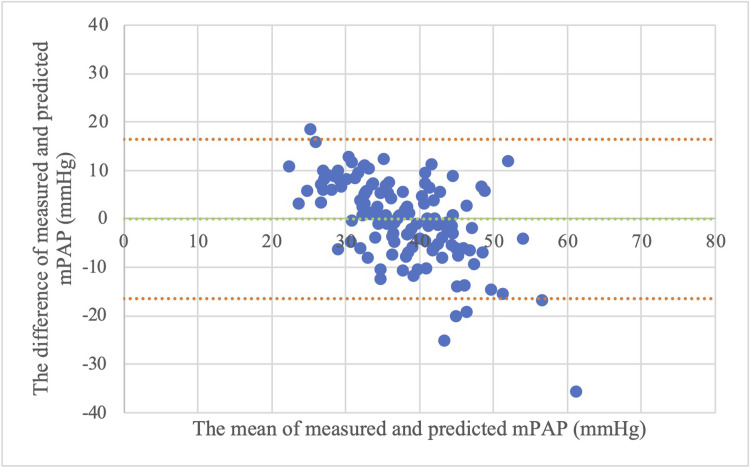
Bland-Altman plot between measured and predicted mPAP (univariable model with only TRPG). Abbreviations: mPAP (mean pulmonary artery pressure), TRPG (tricuspid valve pressure gradient). Note: 95% limits of agreement was -16.5 to 16.5 mmHg.

**Fig 4:**
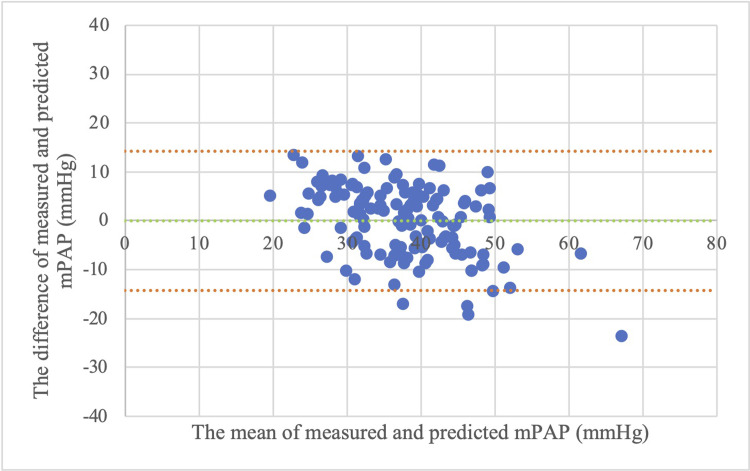
Bland-Altman plot between measured and predicted mPAP (multivariable model with 4 variables). Abbreviation: mPAP (mean pulmonary artery pressure). Note: 95% limits of agreement was -14.2 to 14.2 mmHg.

The prediction results obtained from the conventional model and the optimal multivariable model were plotted on scatter plots and Bland-Altman plots to compare the prediction results visually. Figs 1 and 2 show the scatter plots between the predicted and measured values of mPAP using the conventional and optimal multivariable models, respectively. Figs 1 and 2 show that the prediction results were more accurate in the optimal multivariable model than in the conventional model. Figs 3 and 4 show the Bland-Altman plots between the predicted and measured values of mPAP using the conventional and optimal multivariable models, respectively. Figs 3 and 4 do not show any apparent fixed bias. Fig 3 of the conventional model shows a mild proportional bias. 95% limits of agreement of the conventional model and the optimal multivariable model were -16.5 to 16.5 mmHg and -14.2 to 14.2 mmHg, respectively. The AIC and BIC of the two models were as follows: the optimal multivariable model, 934.1 and 948.7; the conventional model, 968.6 and 974.4. Thus, prediction accuracy was better in the optimal multivariable model than in the conventional model.

**Fig 5:**
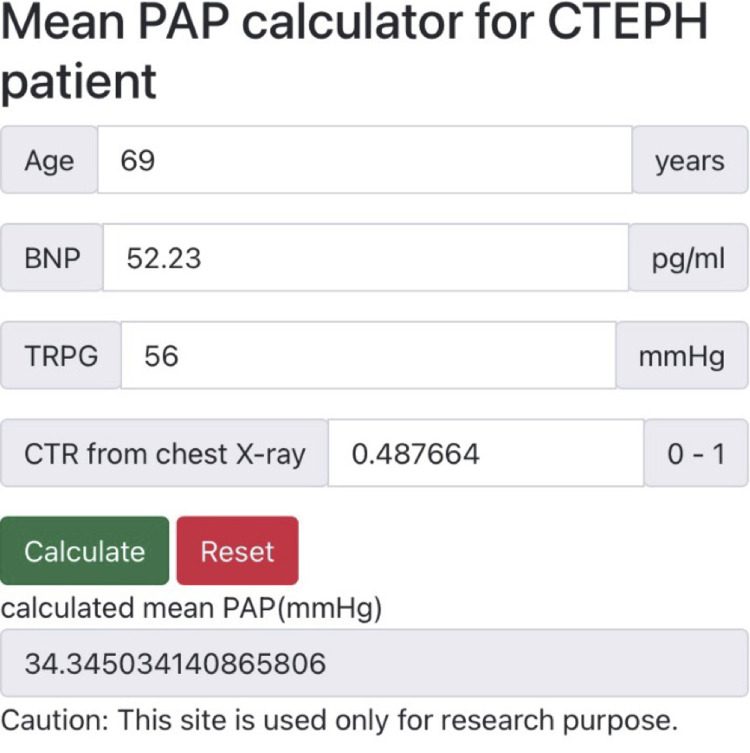
Screenshot of website. Note: This screenshot was obtained using a smartphone.

To make our optimal multivariable model for predicting mPAP publicly available, our website is available at https://benzenedog.github.io/predicting-pulmonary-artery-pressure/. Fig 5 shows a screenshot of the website where the mPAP is predicted on a smartphone. In addition, our source code of this study is available at https://github.com/benzenedog/Regression-of-mPAP-of-CTEPH.

## Discussion

In our study, 136 patients diagnosed with CTEPH at our institution were included to construct a prediction model for mPAP. To construct the prediction models, seven explanatory variables (age, sex, BNP, TRPG, CTR, RSR, and presence of avascular area) were used. We selected the optimal machine learning method and optimal combination of explanatory variables to construct our prediction model. This allowed us to construct the model with higher accuracy than the conventional model, for predicting mPAP. Based on the results of this study, we have validated the hypothesis that the low-invasive and highly accurate assessment and prediction method could be developed using machine learning.

Based on the results of the current study, the linear regression model was the most accurate machine learning method for mPAP prediction in CTEPH. Seven machine learning methods and four AutoML software programs were used to build the model. Among these, the linear model was selected as the optimal method. This result may have been caused by the amount of training data used in the current study. In general, it is possible to build more accurate models than linear models using advanced and complex machine learning methods. However, the generalizability of prediction models tends to decrease in complex models. In our study, the degradation of generalizability was significant in the complex model because the amount of training data was limited. As a result, the linear model was selected as the best machine learning method.

The reason for the performance improvement of our prediction model may be that the selected explanatory variables had a large association with stenosis/occlusion of pulmonary artery, which is the pathogenesis of CTEPH. The TRPG is a metric for disease severity and has been conventionally used to estimate mPAP [8]. BNP reflects the pathophysiology of heart failure and increases in response to ventricular load, suggesting a correlation with mPAP [19]. Our results showed that age, but not sex, had a significant effect.

To improve the prediction accuracy of our model further, new explanatory variables must be added. D-dimer, C-reactive protein (CRP), and transcutaneous arterial oxygen saturation measured using pulse oximetry are possible candidates that are minimally invasive, simple, and inexpensive. Previous studies have shown that D-dimer and CRP are significant prognostic factors in patients with CTEPH [20], and arterial oxygen saturation might be a good predictor of the development of pulmonary arterial hypertension [21]. D-dimer and CRP levels are routinely measured in clinical practice as blood tests, and transcutaneous arterial oxygen saturation can be easily measured using a pulse oximeter. By adding these explanatory variables to our model, further prediction accuracy may be obtained.

Estimated values from our prediction model correlated better with mPAP than those from TRPG. However, we speculate that it is difficult to replace our model with RHC, because of the prediction error of our model. Instead, our model might be a low-risk and low-cost method for assessing trends of the mPAP in CTEPH.

TRPG measured using the Doppler method with echocardiography is a well-known method for estimating mPAP [7]. Other methods of estimating mPAP have been reported, including the construction of an mPAP prediction model using indices measured on computed tomography [22] and magnetic resonance imaging [23–25]. While this study mainly used the TRPG for the model construction, parameters of other modalities such as computed tomography and magnetic resonance imaging were ignored in this study. This is one of the major limitations of this study.

In machine learning, it is typical to assess predictive performance using a separate test set. However, due to the rarity of CTEPH, it is difficult to obtain a sufficient dataset. In this study, we compared machine learning models by averaging the evaluation results from 10 different validation sets to address this issue. We also speculate that there was the possibility of overfitting during model evaluation in this study. However, the linear regression model, which is less prone to overfitting, was found to be the most accurate in this study. If a machine learning model other than linear regression had been optimal, it would have been necessary to prepare a separate test set. However, since the linear regression model was the most accurate and least prone to overfitting, we selected it as the optimal model and did not prepare a separate test set.

The current study had several limitations. First, the data of the current study were collected from our institution (Kobe University Hospital), and external validation was not performed for our model. Because our model is easily available on our website, it is expected that our model will be validated with data from different institutions through our website in the future. Second, the linear model was the best machine learning method in the current study. This is mainly due to the small amount of training data. It is possible that other machine learning methods could be better than the linear model by collecting more training data. However, because of the low disease frequency of CTEPH [3], it might be difficult to collect a large amount of training data. Third, our model can be used only for the patient at the diagnosis of CTEPH. Fourth, our model was constructed from the small-sized training data of patients with CTEPH. If patients without pulmonary hypertension/CTEPH or patients after therapeutic intervention are included in the training data, the independent and identically distributed property required for machine learning methods will not be satisfied and the accuracy of the prediction model will be reduced. Therefore, the mPAP prediction model was constructed with the data of CTEPH at the time of diagnosis. Fifth, our model cannot be applied to patients in which there is no tricuspid regurgitation.

## Conclusion

We constructed a prediction model for mPAP in patients with CTEPH with higher accuracy by performing minimally invasive tests. The prediction performance of our model was improved by selecting the optimal machine learning method and combination of explanatory variables. Our prediction model may be useful for predicting the mPAP in patients with CTEPH.

## Supporting information

S1 Table Multicollinearity of our model. (DOCX)

S1 File Equation and detailed results of the final model. (DOCX)

S1 Fig Results of the normality or homoscedasticity of the errors in the final model. (DOCX)

## References

[1] Humbert M, Kovacs G, Hoeper MM, Badagliacca R, Berger RMF, Brida M, et al. 2022 ESC/ERS Guidelines for the diagnosis and treatment of pulmonary hypertension. Eur Heart J 2022;43:3618–731. 10.1093/EURHEARTJ/EHAC237 36017548

[2] Hoeper MM, Mayer E, Simonneau G, Rubin LJ. Chronic thromboembolic pulmonary hypertension. Circulation 2006;113:2011–20. 10.1161/CIRCULATIONAHA.105.602565 16636189

[3] Jenkins D, Madani M, Fadel E, D’Armini AM, Mayer E. Pulmonary endarterectomy in the management of chronic thromboembolic pulmonary hypertension. European Respiratory Review 2017;26. 10.1183/16000617.0111-2016 28298388 PMC9489144

[4] Kramm T, Mayer E, Dahm M, Guth S, Menzel T, Pitton M, et al. Long-term results after thromboendarterectomy for chronic pulmonary embolism. European Journal of Cardio-Thoracic Surgery 1999;15:579–84. 10.1016/S1010-7940(99)00076-7 10386400

[5] Hoeper MM, Lee SH, Voswinckel R, Palazzini M, Jais X, Marinelli A, et al. Complications of Right Heart Catheterization Procedures in Patients With Pulmonary Hypertension in Experienced Centers. J Am Coll Cardiol 2006;48:2546–52. 10.1016/J.JACC.2006.07.061 17174196

[6] Ullah W, Minalyan A, Saleem S, Nadeem N, Abdullah HM, Abdalla A, et al. Comparative accuracy of non-invasive imaging versus right heart catheterization for the diagnosis of pulmonary hypertension: A systematic review and meta-analysis. IJC Heart and Vasculature 2020;29. 10.1016/j.ijcha.2020.100568 32642551 PMC7334462

[7] Sawada N, Kawata T, Daimon M, Nakao T, Hatano M, Maki H, et al. Detection of pulmonary hypertension with systolic pressure estimated by doppler echocardiography comparison with invasive mean pulmonary artery pressure. Int Heart J 2019;60:836–44. 10.1536/ihj.18-453. 31257329

[8] Fisher MR, Forfia PR, Chamera E, Housten-Harris T, Champion HC, Girgis RE, et al. Accuracy of doppler echocardiography in the hemodynamic assessment of pulmonary hypertension. Am J Respir Crit Care Med 2009;179:615–21. 10.1164/rccm.200811-1691OC 19164700 PMC2720125

[9] Rich JD, Shah SJ, Swamy RS, Kamp A, Rich S. Inaccuracy of doppler echocardiographic estimates of pulmonary artery pressures in patients with pulmonary hypertension: Implications for clinical practice. Chest 2011;139:988–93. 10.1378/chest.10-1269 20864617

[10] Deo RC. Machine learning in medicine. Circulation 2015;132:1920–30. 10.1161/CIRCULATIONAHA.115.001593 26572668 PMC5831252

[11] Yamashita R, Nishio M, Do RKG, Togashi K. Convolutional neural networks: an overview and application in radiology. Insights Imaging 2018;9:611–29. 10.1007/S13244-018-0639-9/FIGURES/15 doi: 10.1007/S13244-018-0639-9/FIGURES/15 29934920 PMC6108980

[12] Satoh T, Kyotani S, Okano Y, Nakanishi N, Kunieda T. Descriptive patterns of severe chronic pulmonary hypertension by chest radiography. Respir Med 2005;99:329–36. 10.1016/j.rmed.2004.08.012 15733509

[13] Mirsadraee M, Nazemi S, Hamedanchi A, Naghibi S. Simple Screening of Pulmonary Artery Hypertension Using Standard Chest X Ray: An Old Technique, New Landmark 2013;12:17–22. PMC4153251 25191469

[14] Chen T, Guestrin C. XGBoost: A Scalable Tree Boosting System. Proceedings of the 22nd ACM SIGKDD International Conference on Knowledge Discovery and Data Mining n.d. 10.1145/2939672.

[15] GitHub—microsoft/FLAML: A fast library for AutoML and tuning. n.d. https://github.com/microsoft/FLAML (accessed February 24, 2022).

[16] TPOT n.d. http://epistasislab.github.io/tpot/ (accessed February 24, 2022).

[17] auto-sklearn—AutoSklearn 0.14.4 documentation n.d. https://automl.github.io/auto-sklearn/master/ (accessed February 24, 2022).

[18] AutoKeras n.d. https://autokeras.com/ (accessed February 24, 2022).

[19] Warwick G, Thomas PS, Yates DH. Biomarkers in pulmonary hypertension. European Respiratory Journal 2008;32:503–12. 10.1183/09031936.00160307 18669790

[20] Skoro-Sajer N, Gerges C, Gerges M, Panzenböck A, Jakowitsch J, Kurz A, et al. Usefulness of thrombosis and inflammation biomarkers in chronic thromboembolic pulmonary hypertension—sampling plasma and surgical specimens. Journal of Heart and Lung Transplantation 2018;37:1067–74. 10.1016/j.healun.2018.04.003 29802084

[21] Park HK, Shin HJ, Park YH, Ma BG. The importance of preoperative oxygen saturation as a predictor of pulmonary arterial hypertension after surgery of atrial septal defects. Interact Cardiovasc Thorac Surg 2016;23:424–30. 10.1093/icvts/ivw162 27222001

[22] Li M, Wang S, Lin W, Li J, Wang C, Chen H, et al. Cardiovascular parameters of chest CT scan in estimating pulmonary arterial pressure in patients with pulmonary hypertension. Clin Respir J 2018;12:572–9. 10.1111/CRJ.12564 27696745

[23] Reiter U, Kovacs G, Reiter C, Kräuter C, Nizhnikava V, Fuchsjäger M, et al. MR 4D flow-based mean pulmonary arterial pressure tracking in pulmonary hypertension. Eur Radiol 2021;31:1883–93. 10.1007/S00330-020-07287-6 32974687 PMC7979582

[24] Ohno Y, Koyama H, Yoshikawa T, Nishio M, Matsumoto S, Matsumoto K, et al. Contrast-enhanced multidetector-row computed tomography vs. Time-resolved magnetic resonance angiography vs. contrast-enhanced perfusion MRI: Assessment of treatment response by patients with inoperable chronic thromboembolic pulmonary hypertension. Journal of Magnetic Resonance Imaging 2012;36:612–23. 10.1002/JMRI.23680 22566188

[25] Nogami M, Ohno Y, Koyama H, Kono A, Takenaka D, Kataoka T, et al. Utility of phase contrast MR imaging for assessment of pulmonary flow and pressure estimation in patients with pulmonary hypertension: Comparison with right heart catheterization and echocardiography. Journal of Magnetic Resonance Imaging 2009;30:973–80. 10.1002/JMRI.21935 19856412

