# Supplementary material for: Development and web deployment of prediction model for pulmonary arterial pressure in chronic thromboembolic pulmonary hypertension using machine learning
Source: PLoS One. 2024 Apr 5;19(4):e0300716. doi: 10.1371/journal.pone.0300716 (PMC10997056; doi:10.1371/journal.pone.0300716)
Supplement: S1 Table — (DOCX) [file pone.0300716.s001.docx]

**Supporting information**

**S1 Table**

Multicollinearity of our model

In summary, there was no multicollinearity in the explanatory variables of our optimal model. The correlation coefficient and variance inflation factor (VIF) for the explanatory variables are summarized as the following Tables.

| combination of explanatory variables | correlation coefficient |
| --- | --- |
| age, log BNP | 0.154 |
| age, TRPG | -0.077 |
| age, log CTR | 0.148 |
| log BNP, TRPG | 0.427 |
| log BNP, log CTR | 0.494 |
| TRPG, log CTR | 0.088 |

| explanatory variables | VIF |
| --- | --- |
| age | 1.055 |
| log BNP | 1.684 |
| TRPG | 1.281 |
| log CTR | 1.361 |
