# Supplementary material for: Development and web deployment of prediction model for pulmonary arterial pressure in chronic thromboembolic pulmonary hypertension using machine learning
Source: PLoS One. 2024 Apr 5;19(4):e0300716. doi: 10.1371/journal.pone.0300716 (PMC10997056; doi:10.1371/journal.pone.0300716)
Supplement: S1 File — (DOCX) [file pone.0300716.s002.docx]

**Supporting information**

**S1 File**

Equation and detailed results of the final model

$$mPAP=-0.2758\times age+0.2316\times TRPG+1.6929\times\log\left( BNP \right)+11.3598\times\log\left( CTR \right)+42.0532$$

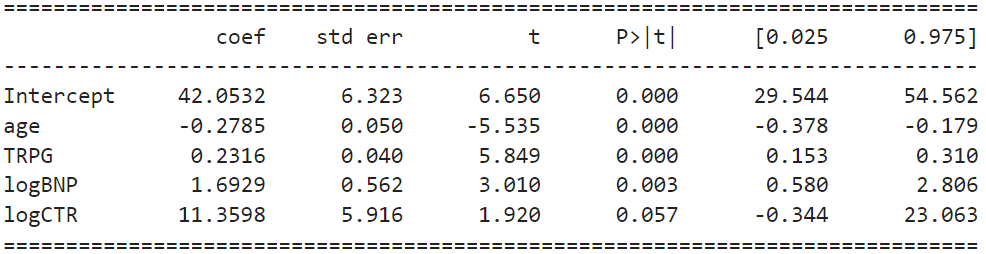


Note: These results were obtained using the statsmodels package in Python.
