# Supplementary material for: Development and web deployment of prediction model for pulmonary arterial pressure in chronic thromboembolic pulmonary hypertension using machine learning
Source: PLoS One. 2024 Apr 5;19(4):e0300716. doi: 10.1371/journal.pone.0300716 (PMC10997056; doi:10.1371/journal.pone.0300716)
Supplement: S1 Fig — (DOCX) [file pone.0300716.s003.docx]

**Supporting information**

**S1 Figure**

Results of the normality or homoscedasticity of the errors in the final model


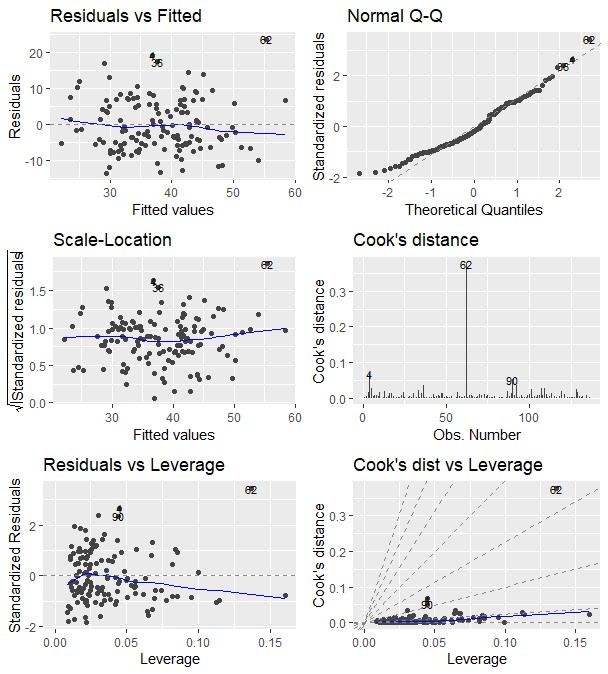


There were no significant issues with the normality or homoscedasticity of the errors.
